# Transcriptional analysis of the conidiation pattern shift of the entomopathogenic fungus *Metarhizium acridum* in response to different nutrients

**DOI:** 10.1186/s12864-016-2971-0

**Published:** 2016-08-09

**Authors:** Zhenglong Wang, Kai Jin, Yuxian Xia

**Affiliations:** 1 Genetic Engineering Research Center, School of Life Sciences, Chongqing University, Chongqing, 400045 People’s Republic of China; 2 Chongqing Engineering Research Center for Fungal Insecticide, Chongqing University, Chongqing, 400045 People’s Republic of China; 3 Key Laboratory of Gene Function and Regulation Technologies under Chongqing Municipal Education Commission, Chongqing University, Chongqing, 400045 People’s Republic of China

**Keywords:** Conidiation pattern shift, Normal and microcycle conidia, *Metarhizium acridum*, Pathway analysis

## Abstract

**Background:**

Most fungi, including entomopathogenic fungi, have two different conidiation patterns, normal and microcycle conidiation, under different culture conditions, eg, in media containing different nutrients. However, the mechanisms underlying the conidiation pattern shift are poorly understood.

**Results:**

In this study, *Metarhizium acridum* undergoing microcycle conidiation on sucrose yeast extract agar (SYA) medium shifted to normal conidiation when the medium was supplemented with sucrose, nitrate, or phosphate. By linking changes in nutrients with the conidiation pattern shift and transcriptional changes, we obtained conidiation pattern shift libraries by Solexa/Illumina deep-sequencing technology. A comparative analysis demonstrated that the expression of 137 genes was up-regulated during the shift to normal conidiation, while the expression of 436 genes was up-regulated at the microcycle conidiation stage. A comparison of subtractive libraries revealed that 83, 216, and 168 genes were related to sucrose-induced, nitrate-induced, and phosphate-induced conidiation pattern shifts, respectively. The expression of 217 genes whose expression was specific to microcycle conidiation was further analyzed by the gene expression profiling via multigene concatemers method using mRNA isolated from *M. acridum* grown on SYA and the four normal conidiation media. The expression of 142 genes was confirmed to be up-regulated on standard SYA medium. Of these 142 genes, 101 encode hypothetical proteins or proteins of unknown function, and only 41 genes encode proteins with putative functions. Of these 41 genes, 18 are related to cell growth, 10 are related to cell proliferation, three are related to the cell cycle, three are related to cell differentiation, two are related to cell wall synthesis, two are related to cell division, and seven have other functions. These results indicate that the conidiation pattern shift in *M. acridum* mainly results from changes in cell growth and proliferation.

**Conclusions:**

The results indicate that *M. acridum* shifts conidiation pattern from microcycle conidiation to normal conidiation when there is increased sucrose, nitrate, or phosphate in the medium during microcycle conidiation. The regulation of conidiation patterning is a complex process involving the cell cycle and metabolism of *M. acridum*. This study provides essential information about the molecular mechanism of the induction of the conidiation pattern shift by single nutrients.

**Electronic supplementary material:**

The online version of this article (doi:10.1186/s12864-016-2971-0) contains supplementary material, which is available to authorized users.

## Background

Conidia (spores) are the beginning and end of the differentiation process in the lifecycle of fungi [1, 2], and they play important roles in reproduction and survival [2]. Most filamentous fungi have two conidiation patterns: normal and microcycle conidiation [3]. Normal conidiation is the most common reproductive mode of filamentous fungi [4], and microcycle conidiation is a survival mechanism under stress conditions, whereby the normal lifecycle is bypassed [3, 5–8]. To date, microcycle conidiation has been described in more than 100 fungal species [3, 5, 7], and it has been divided into four basic categories based on the morphological characteristics of conidia [9].

The conidiation patterns can be shifted from normal to microcycle conidiation under various conditions, such as high and/or low temperature [6, 10, 11], high and/or low pH [12, 13], high salt concentration [14], and the presence of certain nutrients [7, 10, 15, 16]. Nutrients are the most common factors that affect fungal conidiation patterns. In *Colletotrichum gloeosporioides*, microcycle conidiation occurs in substrate-limited liquid cultures [17]. In *Beauveria bassiana*, microcycle conidiation is observed in the absence of a carbon source in the basal medium [15]. In *Aspergillus flavus*, exogenous putrescine inhibits microcycle conidiation and induces mycelial development [18]. In *Neurospora intermedia*, microcycle conidiation occurs under low sugar and nitrogen conditions [19]. The culture conditions for normal and microcycle conidiation are different, and even a subtle change in culture conditions can cause a substantially different conidiation pattern. The conidiation pattern shift in response to nutrients may be regulated by some sensors and pathways [20]. However, the molecular mechanisms of the conidiation pattern shift in response to nutrients have not been elucidated.

The conidia of entomopathogenic fungi are formulated as myco-insecticides [21–23]. *Metarhizium acridum* is a model system for entomopathogenic fungi, and it is widely used for locust control in Africa, Asia, and Australia [24–26]. The entomopathogenic fungus *M. acridum* displays two conidiation patterns: normal conidiation on 1/4 strength Sabouraud’s dextrose agar medium (1/4 SDAY), but microcycle conidiation on sucrose yeast extract (SYA) medium [27]. In the present study, the effects of single nutrients on the conidiation pattern of *M. acridum* were investigated by the addition of 7.5 % sucrose (sucrose-rich (SR) medium), 0.75 % nitrate (nitrate-rich (NR) medium) or 0.25 % phosphate (phosphate-rich (PR) medium) to the microcycle conidiation medium (SYA). The results showed that normal conidiation occurred on 1/4 SDAY and the three nutrient-rich media, and conidiophores and normal conidiation occurred 21 h post-inoculation (hpi), while microcycle conidia were produced on SYA medium during this period. The transcripts of *M. acridum* derived from SYA medium, the three nutrient-rich media, and 1/4 SDAY medium were compared. The genes involved in the conidiation pattern shift and the genes involved in the regulation of the conidiation pattern shift in the three nutrient-rich media were identified. Then, the mechanisms of the conidiation pattern shift of *M. acridum* in response to different nutrients were explored.

## Results

### Conidiation pattern shift of *M. acridum* in response to different nutrients

**Fig. 1:**
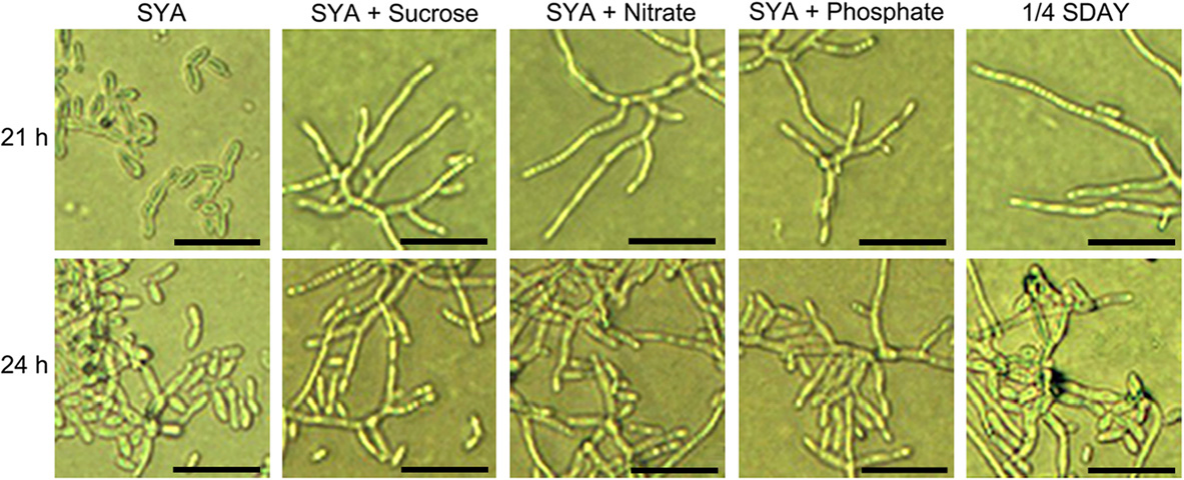
Different conidiation patterns of *M. acridum* CQMa102 on different agar media following incubation at 28 °C. Plates were inverted and photographed (400×). Scale bar = 100 μm

To investigate the effects of single nutrients on the conidiation pattern shift, *M. acridum* was grown on the microcycle conidiation medium (SYA), normal conidiation medium (1/4 SDAY), and SYA medium supplemented with sucrose, nitrate, or phosphate. On 1/4 SDAY, SR, NR, and PR media, conidiophores appeared without conidia at 21 hpi. Normal conidiation took place after 24 hpi, while microcycle conidia were produced on SYA medium during this period. The morphology of the normal and microcycle conidia differed significantly, with microcycle conidia having a more uniform size than normal conidia (Fig. 1). These results indicate that all the nutrients, including sucrose, nitrate, and phosphate, can influence the conidiation pattern shift and cause morphological changes in the conidia of *M. acridum*.

### Characterization of a digital gene expression (DGE) database

To elucidate the molecular mechanisms of the conidiation pattern shift that was regulated by single nutrients, mRNA derived from *M. acridum* cultured on SYA, SR, NR, PR, and 1/4 SDAY media was used to construct five digital gene expression (DGE) libraries. Approximately 6 million sequence tags, 2 million of which were distinct, were obtained for all five DGE libraries. For each library, more than 60 % of the tags were mapped to the transcription reference database of *M. acridum* [28]. Major characteristics of the libraries are shown in Table 1. The copy number of a tag reflected the mRNA expression level in clean tags, and the distribution of clean tag expression could be used to evaluate the normality of all the data. The distribution of total tags and distinct tags suggests that a small number of mRNAs were highly abundant, but the majority of mRNAs were expressed at low levels, thus meeting the heterogeneity law of gene expression (Additional file 1). The results indicated that our sequencing data are credible and suitable for further analysis.

**Table 1:** Major characteristics of all the DGE libraries

Category	Parameter	Value for conidiation library				
		1/4SDAY	SYA	C-source rich	N-source rich	P-source rich
Raw tag	Total no. of tags	6,183,315	5,825,445	6,065,919	6,013,042	6,248,039
	No. of distinct tags	281,640	270,988	261,287	310,019	284,707
Clean tag	Total no. of tags	6,002,447	5,654,488	5,893,027	5,810,564	6,062,673
	No. of distinct tags	114,124	111,004	102,142	118,874	113,434
Unambiguous tag-mapped genes	No. of genes	6340	6520	5854	6448	6371
	% of reference genes	62.85	64.63	58.03	63.92	63.15

**Fig. 2:**
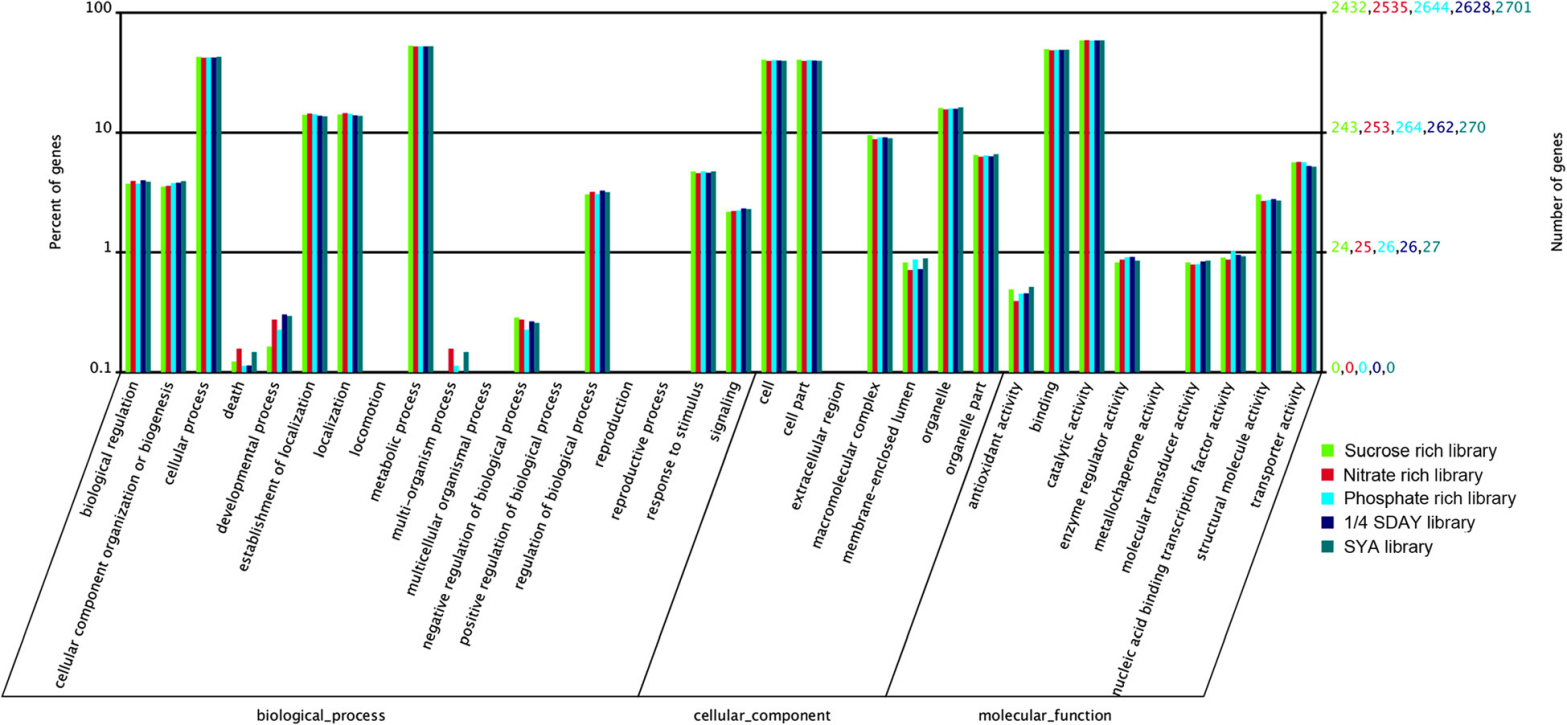
Histogram of GO classification of putative gene functions from the five libraries. The functions of identified genes cover three main categories: biological process, cellular component, and molecular function. The right *y*-axis indicates the number of genes in a category. The left *y*-axis indicates the percentage of a specific category of genes in a main category. GO analysis showed that the distributions of gene functions for the five libraries are similar

Using a gene ontology (GO) analysis of the genes mapped in the reference database of the *M. acridum* genome, we constructed a particular GO hierarchy of (i) biological process, (ii) cellular component, and (iii) molecular function for each library (http://wego.genomics.org.cn/cgi-bin/wego/index.pl) (Fig. 2). For molecular function, the most significant enrichment was observed among various binding genes (GO: 0005488) and catalytic activity genes (GO: 0003824). For biological process, the most significant enrichment was observed among cellular process (GO: 0009987) and metabolic process (0008152). For cellular component, about 71 % of the differentially expressed genes (DEGs) were found to be involved in “cell” structure; these included genes related to the plasma membrane and external encapsulating structures, such as the cell wall and cell envelope (Additional file 2).

### DEGs of *M. acridum* during normal and microcycle conidiation

**Fig. 3:**
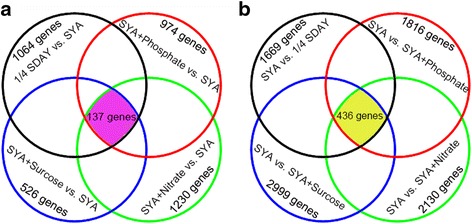
Screening of differentially expressed genes involved in conidiation pattern shift. **a** The genes were up-regulated during the normal conidiation. The boundaries of each subtractive library are delimited by specific colors: 1/4SDAY vs. SYA subtractive library (*black*); SYA+Phosphate vs. SYA subtractive library (*red*); SYA+Nitrate vs. SYA  subtractive library (*green*); SYA+Sucrose vs. SYA  subtractive library (*blue*). **b** The genes were up-regulated during the microcycle conidiation. The boundaries of each subtractive library are delimited by specific colors: SYA vs. 1/4SDAY subtractive library (*black*);  SYA vs. SYA+Phosphate subtractive library (*red*); SYA vs. SYA+Nitrate subtractive library (*green*); SYA vs. SYA+Sucrose subtractive library (*blue*)

To elucidate the molecular mechanism of the conidiation pattern shift, genes whose expression was up-regulated genes during normal and microcycle conidiation were screened by constructing four subtractive libraries based on the five DGE libraries. A comparison of the four subtractive libraries revealed that the expression of 137 genes was up-regulated in the four normal conidiation media (Fig. 3a), and the expression of 436 genes was up-regulated in the microcycle conidiation medium (SYA) (Fig. 3b). Among the 137 genes whose expression was up-regulated during the normal conidiation stage, there were three transcription factors, seven absorption- and transportation-related genes, including one amino acid transporter, two major facilitator superfamily (MFS) transporters, two ATP-binding cassette (ABC) transporters, one sulfate transporter, and one oligopeptide transporter, six stress-related genes, including two cytochrome P450 genes, and four nutrient and energy metabolism-related genes, such as one glycolysis-related gene, one tricarboxylic acid (TCA)-cycle-related genes, and two phosphorylation-related genes (Additional file 3). A GO analysis showed that the genes are mainly involved in catalytic activity, transporter activity, and binding function processes (Additional file 2). These genes are mainly involved in amino acid metabolism, cell growth and death, energy metabolism, lipid metabolism, metabolism of terpenoids and polyketides, xenobiotics biodegradation and metabolism, biosynthesis of secondary metabolites, and carbohydrate metabolism pathways (Additional file 4). The results indicate that normal conidiation in *M. acridum* is a complex process that involves multiple genes and biological processes.

Among the 436 genes whose expression was up-regulated during microcycle conidiation, there were 16 transcription factors, including five zinc finger protein (ZFP) transcription factors, one basic leucine zipper domain (bZIP) family transcription factor, and one helix-loop-helix (HLH) transcription factors, five mitogen-activated protein (MAP) kinases, 15 absorption- and transportation-related genes, including three carbohydrate and water reabsorption genes, six RNA transport proteins, five stress-related genes, including two cytochrome P450 genes, two peroxidase genes, and one glutathione-disulfide reductase gene, and 13 nutrient and energy metabolism-related genes, including four glycolysis-related genes, three TCA-cycle-related genes, and six phosphorylation-related genes (Additional file 5). A GO analysis revealed that these genes are mainly involved in catalytic activity, transporter activity, and binding function processes (Additional file 1: Table S1). These genes are mainly involved in amino acid metabolism, microbial metabolism in diverse environments, carbohydrate metabolism, cell growth and death, energy metabolism, lipid metabolism, xenobiotics biodegradation and metabolism, metabolism of terpenoids and polyketides, nucleotide metabolism, replication and repair, signal transduction, translation, transcription, biosynthesis of secondary metabolites, glycan biosynthesis and metabolism, transport and catabolism, and metabolism of cofactors and vitamins pathways (Additional file 4). Many genes whose expression was up-regulated during microcycle conidiation encode proteins that function in cell division, cell proliferation, cell wall formation, and cytoskeletal rearrangement, including a tyrosine-protein phosphatase [29], a transcriptional coactivator [30], a zinc knuckle domain protein [31], a serine-type carboxypeptidase [32], sedoheptulose-1, 7-bisphosphatase [33], a catalase [34, 35], cytochrome P450 [36], a mannan endo-1, 6-α-mannosidase-like protein [37, 38], an actin-associated protein [39], and a HLH transcription factor [40], suggesting that these up-regulated genes play a role in microcycle conidiation. Interestingly, members of the normal conidiation FluG pathway, including *snaD*, *GNAT*, *fluG*, *pkaA* [41], *fadA* [42], and *gasA* [43], were up-regulated during microcycle conidiation (Additional file 5).

These results indicate that genes related to both normal and microcycle conidiation are mainly involved in amino acid metabolism, cell growth and death, energy metabolism, lipid metabolism, metabolism of terpenoids and polyketides, translation pathways, and other pathways (Additional file 4). These pathways participate in cell proliferation, cell development, cell cycle, and cytoskeletal rearrangement processes. The common pathways in the conidiation pattern shift indicate that normal and microcycle conidiation have similar developmental processes and can be regulated through some common pathways, eg, the FluG pathway. However, compared with normal conidiation, microcycle conidiation involved two more pathways, more genes in 10 pathways (Additional file 4), and higher gene expression in common pathways, such as the FluG pathway. These genes and pathways might be related to the shift between normal and microcycle conidiation.

### Genes involved in the conidiation pattern shift related to different nutrients

**Fig. 4:**
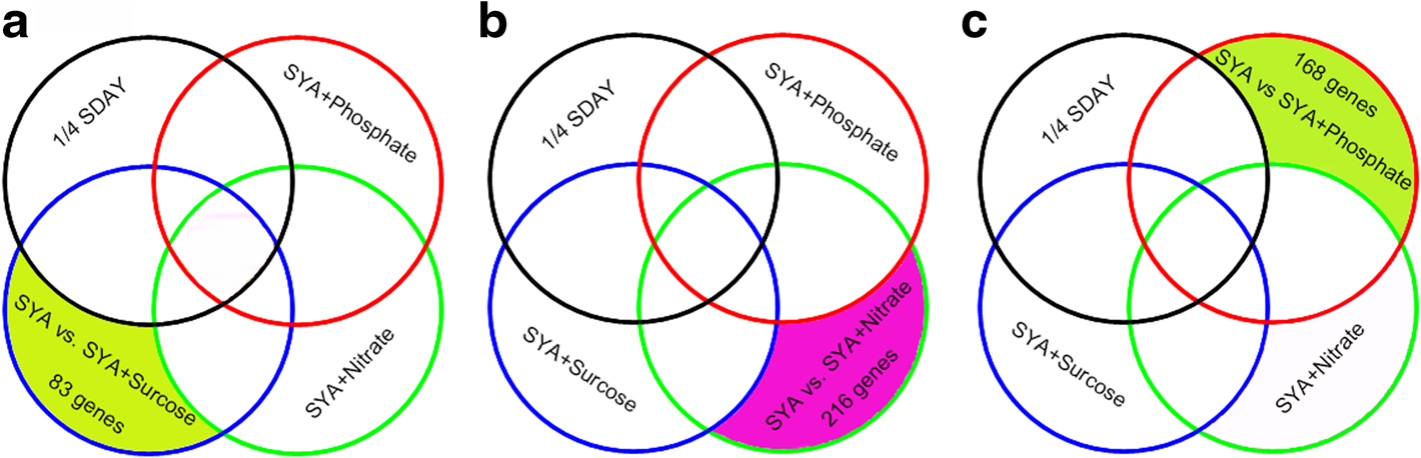
Screening of differentially expressed genes involved in conidiation pattern shift when related to different special nutrients. **a** The differentially expressed genes invovled in conidiation pattern shift were regulated by sucrose. **b** The differentially expressed genes invovled in  conidiation pattern shift were regulated by nitrate. **c** The differentially expressed genes invovled in  conidiation pattern shift were regulated by phosphate. The non-simple Venn diagram shows unique and overlapping sets of transcripts between the libraries. The boundaries of each library are delimited by specific colors: 1/4 SDAY medium library (*black*); SYA+Phosphate medium library (*red*); SYA+Nitrate medium library (*green*); SYA+Sucrose medium library (*blue*)

Because the conidiation pattern of *M. acridum* could be regulated by single nutrients, including sucrose, nitrate, and phosphate, we constructed three subtractive libraries to screen for genes that are specifically expressed in the conidiation pattern shift in response to these nutrients. The three subtractive libraries were compared individually with the genes expressed in 1/4 SDAY medium and the other two nutrient-rich media libraries, which showed that 83, 216, and 168 genes were specifically expressed on SR, NR, and PR media, respectively, indicating that they are involved in the regulation of the conidiation pattern shift by these nutrients (Fig. 4). Among the 83 sucrose-regulated genes, the expression of 75 genes was up-regulated, and the expression of eight genes was down-regulated (Additional file 6). Among them, there were four transcription factors, five stress-related genes, including three cytochrome P450 genes, one lipoxygenase, and one phytanoyl-CoA dioxygenase, six metabolism-related genes, including three glycolysis-related genes, two binding proteins, and one protein tyrosine phosphatase, and two genes involved in the cell cycle process. A GO analysis found that most of the genes played roles in catalytic activity, oxidoreductase activity, hydrolase activity, and metal ion binding (Additional file 6). A pathway analysis found that these DEGs are mainly involved in amino acid metabolism, biosynthesis of secondary metabolites, lipid metabolism, xenobiotics biodegradation and metabolism, metabolism of cofactors and vitamins, and transcription pathways (Additional file 7). The results indicate that sucrose could facilitate cell growth and inhibit sporulation by changing metabolic pathways.

With respect to the nitrate-rich medium, 216 specifically expressed genes were screened in the target libraries. Of these, the expression of 203 genes was up-regulated, and the expression of 13 genes was down-regulated (Additional file 8). Among these, there were seven transcription factors, including four zinc finger transcription factors and one GATA-binding transcription factor, three stress-related genes, including two cytochrome P450 genes and one dioxygenase, 41 metabolism-related genes, including three binding proteins and one protein tyrosine phosphatase, and five genes involved in the cell cycle process. A pathway analysis found that these genes are mainly involved in amino acid metabolism, carbohydrate metabolism, cell growth and death, lipid metabolism, xenobiotics biodegradation and metabolism, nucleotide metabolism, transport and catabolism pathways, and microbial metabolism in diverse environments pathways (Additional file 7). A GO analysis found that most of the genes had roles in catalytic activity, protein kinase activity, transporter activity, transferase activity, hydrolase activity, and adenyl ribonucleotide binding. The results showed that at a high nitrate level, the expression of catalytic-, hydrolase-, transferase-, and ribonucleotide-binding-related genes was up-regulated. A reasonable explanation is that nitrogen affects cell morphology by controlling amino acid metabolism.

For the phosphate-rich medium, 168 specifically expressed genes were filtered in the target libraries. Of these, the expression of 151 genes was up-regulated, and the expression of 17 genes was down-regulated (Additional file 9). Among these, there were five transcription factors, including three zinc finger transcription factors, seven stress-related genes, including two cytochrome P450 genes and three dioxygenases, 24 metabolism-related genes, including two TCA-cycle-related genes, one phosphorylation-related gene, five binding proteins, and three phosphatases, and two cell cycle genes. A pathway analysis found that the genes are mainly involved in amino acid metabolism, carbohydrate metabolism, cell growth and death, lipid metabolism, xenobiotics biodegradation and metabolism, transport and catabolism, and microbial metabolism in diverse environment pathways (Additional file 7). A GO analysis found that most of the genes are involved in catalytic activity, nucleic acid binding, protein kinase activity, transferase activity, adenyl ribonucleotide binding, ATPase activity, and peptidase activity. The results showed that phosphate might affect cell division, proliferation, and differentiation by controlling the formation of ATP and affecting the cytoskeleton.

Carbon, nitrogen, and phosphorus are basic components of the cell. They are constituents of nucleic acids, sugar-phosphate backbones, and phospholipid bilayers, and they are required for cell division and membrane formation [44]. However, the conidiation pattern shifts in response to these nutrients were controlled by different genes, some of which played roles in the same pathway. MAP kinase [45], the origin recognition complex subunit [46], and a serine/threonine protein kinase [47] are located in the cell growth and death pathway, and they played roles in the conidiation pattern shift in response to sucrose, nitrate, and phosphate, respectively. The conidiation pattern shift in response to nutrients involved different pathways. Exo-beta-D-glucosaminidase is involved in the sucrose-induced conidiation pattern shift, and it has an effect on hyphal growth at low sugar concentrations [48]. Acyltransferase is involved in the nitrate-induced conidiation pattern shift, and it participates in the composition of the membrane at high nitrogen concentrations [49]. L-asparaginase is involved in the phosphate-induced conidiation pattern shift, and it has an effect on growth under certain phosphate concentrations [50, 51]. These results indicate that carbon, nitrogen, and phosphorus influence the fungal conidiation pattern by perturbing the cell cycle, nutrient metabolism, and related pathways of *M. acridum*.

### DEGs involved in conidiation were confirmed by the gene expression profiling via multigene concatemers (MgC-GEP) method

To confirm the reliability of the DEGs related to conidiation patterns and their shifts, the expression profiles of the 217 genes that were specific to microcycle conidiation were further analyzed by the MgC-GEP method [52] using the mRNA of *M. acridum* grown on SYA and the four normal conidiation media. One hundred and eighty genes were found using primer pairs targeting the 217 genes, and the expression of 142 genes was confirmed to be up-regulated on SYA medium in comparison with the four normal conidiation media (Additional files 10 and 11). Of these 142 genes, 101 genes encode hypothetical proteins or proteins of unclear function, and only 41 genes encode proteins with putative functions. Of these 41 genes, 18 are related to cell growth, 10 are related to cell proliferation, three are related to the cell cycle, three are related to cell differentiation, two are related to cell wall synthesis, two are related to cell division, and seven have other functions (Table 2). These results indicate that the conidiation pattern shift in *M. acridum* mainly results from changes in cell growth and proliferation.

**Table 2:** Functions of some microcycle conidiation-relate genes screened by Gene Expression profiling via Multigene Concatemers

Functions	Gene ID	Name	References
cell growth	EFY89451	catalase	[34]
	EFY88880	glycosyl hydrolase, family 18, putative	[66]
	EFY89474	isoamyl alcohol oxidase, putative	[67]
	EFY91609	C6 transcription factor	[68]
	EFY90847	thiamine pyrophosphokinase	[69]
	EFY90535	serine-type carboxypeptidase	[32]
	EFY85230	transcription factor ATF2	[70]
	EFY86579	cytochrome P450 3A17	[36]
	EFY84442	amino acid transporter, putative	[71]
	EFY89681	LysM domain-containing protein	[72]
	EFY89664	zinc knuckle domain protein	[31]
	EFZ01737	acetyltransferase	[73]
	EFY91481	glycerophosphoryl diester phosphodiesterase family protein	[74]
	EFY86138	ERV2 protein-like protein	[75]
	EFY86028	sedoheptulose-1,7-bisphosphatase	[76]
	EFY89284	putative ZIP zinc transporter	[77]
	EFY89276	histone acetyltransferase Spt10	[78]
	EFY96753	integral membrane protein	[79]
cell differentiation	EFY96670	late sexual development protein	[80]
	EFY88310	sister chromatid cohesion protein Ctf8, putative	[81]
	EFY89451	catalase	[34]
cell proliferation	EFY89276	histone acetyltransferase Spt10	[78]
	EFY96753	integral membrane protein	[79]
	EFY91612	exonuclease III	[82]
	EFY91973	exosome complex exonuclease Rrp	[83]
	EFY91719	putative transcriptional coactivator HFI1	[30]
	EFY89770	HLH transcription factor (Hpa3), putative	[40]
	EFY92461	Ser/Thr protein phosphatase	[84]
	EFY92813	high mobility group protein	[85]
	EFY89768	alcohol dehydrogenase, putative	[86]
	EFY89823	potassium channel	[87]
cell wall synthesis	EFY92600	dihydrodipicolinate synthase, putative	[88]
	EFY89521	hydrophobin	[89]
cell cycle	EFY88685	checkpoint protein kinase, putative	[90]
	EFY92449	putative xylanase 3	[91]
	EFY90895	tyrosine-protein phosphatase CDC14	[29]
cell division	EFY90773	serine/threonine-protein kinase hal4	[92]
	EFY90261	integral membrane protein	[93]
related to heme synthesis	EFY87119	porphobilinogen deaminase	[94]
related to sucrose uptake	EFY88112	alpha-amylase 1	[95]
inhibition of hyphal extension	EFY87853	chitinase	[96]
related to metabolism	EFY84464	LysR family regulatory protein, putative	[97]
related to metabolism	EFY84562	NADP-dependent alcohol dehydrogenase C	[98]
biosynthesis	EFY85651	acetolactate synthase	[99]

## Discussion

Most fungi have two different conidiation patterns, and the mechanisms of normal conidiation have been well studied in most industrial and model species [1, 2]. In *Aspergillus nidulans*, *snaD* encodes a spindle pole body protein, which is a positive factor in the conidiation process, by promoting the nucleation of tip cells and cell division [53]. In *Neurospora crassa*, *GNAT* negatively regulates conidiation through a cAMP-dependent pathway on solid media, and deletion of *GNAT* leads to decreased aerial hyphal height and premature conidiation [54]. In *M. acridum*, *FKS* is involved in cell wall integrity and conidiation [55]. However, the mechanisms of the conidiation pattern shift are not clear.

Previous research found that *M. acridum* produces microcycle conidia on insect cadavers, while producing normal conidia on PDAY medium [16], suggesting that microcycle conidia maybe produced under nutritive stress. In the present study, microcycle conidiation of *M. acridum* was shifted to normal conidiation after adding single nutrients to SYA medium. This phenotypic change is similar to the conidiation pattern shift in *B. bassiana* [14] and *A. flavus* [17]. Our results revealed that nutrients can cause the conidiation pattern shift in *M. acridum*. However, the underlying mechanisms are far from clear.

To understand the mechanism of the conidiation pattern shift, we used a statistical comparison analysis to obtain 1040 DEGs that are involved in the conidiation pattern shift. These genes are involved in catalytic activity, cellular transport, cell cycle, developmental processes, signal transduction, stress responses, and metabolic processes. The results indicated that a very complex molecular network is involved in the conidiation pattern shift.

The conidiation pattern shift can be induced in some fungi by different nutrients [7, 14, 56]. Therefore, some genes that are regulated by nutrients might be involved in the conidiation pattern shift. The glucose level-sensing gene *snf*3 [57] and the nitrogen-starvation gene *glnA* [58] were detected in our conidiation pattern shift-related gene library. The results indicate that exogenous nutrients affected the conidiation pattern shift by controlling the expression of nutrient-related genes. Our research identified pathways and molecular mechanisms for the conidiation pattern shift that are regulated by a single nutrient.

Previous studies have indicated that microcycle conidiation can produce more spores in a shorter time compared with normal conidiation [59, 60]. Moreover, the spores formed by microcycle conidiation are more uniform in size compared with those formed by normal conidiation [27, 61]. This means that the conidiation pattern shift may be associated with cell growth, cell division, cytoskeletal rearrangement, and energy metabolism. *snaD* and *GNAT* are likely to be involved in this process. In *A. nidulans*, *snaD* affects conidiation by promoting the nucleation of tip cells and cell division [53]. In *N. crassa*, *GNAT* induces the conidiation pattern shift by reducing intracellular cAMP levels [54]. Our results indicate that the conidiation pattern shift of *M. acridum* is consistent with those of industrial and model species. *pcvA* [62], *gasA* [63], *fadA* [43], *GNAT* [54] and *pkaA* [42] were reported to be specific, negative regulators of conidiation, but their transcription levels were higher during microcycle conidiation than normal conidiation. It may be that: (1) these genes are related to nutrient starvation, and on nutrient-rich media, their function is inhibited by nutrients, which results in normal conidiation; and (2) the transcription levels of these genes may be related to spore production.

In summary, there are differences in the morphological and genetic features between the two conidiation patterns. However, little information has been reported about the process of the conidiation pattern shift. The present study used Solexa/Illumina deep-sequencing technology to show that a large number of DEGs are associated with the conidiation pattern shift in *M. acridum* in the presence of single nutrients. Further studies could confirm these DEGs using transgenic technology to enhance the yields of biopesticides. This study also provides a template for future investigations of the conidiation pattern shift in fungi, and it offers hints about the molecular mechanism of conidiation regulation by different nutrients.

## Conclusions

Our results indicate that the conidiation pattern shift is a complex biological process in which the cell cycle and metabolism of *M. acridum* are perturbed by sucrose, nitrate, and phosphate. This research provides essential information about the molecular mechanism of the conidiation pattern shift that is induced by single nutrients.

## Methods

### Fungal strain and culture conditions

The *M. acridum* strain CQMa102 used in this study is a locust-specific strain that was isolated by the Genetic Engineering Research Center of Chongqing University and used in a previous study [25]. Conidia were harvested after the fungus was cultured on 1/4 SDAY medium (SDAY, 1 % dextrose, 0.25 % mycological peptone, 2 % agar and 0.5 % yeast extract, w/v) at 28 °C for 12–15 d.

SYA medium was used for microcycle conidiation under previously described conditions [64]. Sucrose-, nitrate-, and phosphate-rich media, which were used for normal conidiation, had the same composition as SYA medium, except the amount of sucrose, nitrate and phosphate increased to 75, 7.5, and 2.5 g/L, respectively.

### Library preparation and sequencing

After incubation for 21 h at 28 °C, the fungal cell mass was harvested by centrifugation and washed with sterile double-distilled H_2_O. Total RNA was extracted from the fungal cell mass using Trizol reagent (Invitrogen, Carlsbad, CA, USA) according to the manufacturer’s instructions. Oligo(dT) magnetic beads were used to purify mRNA from 6 μg of total RNA, and an oligo(dT) primer was used to synthesize first- and second-strand cDNAs. Sequencing was performed on a High-Seq2000 platform (Illumina, San Diego, CA, USA). Sequence analysis was conducted as described previously [28].

### Screening of DEGs

Based on the five DGE databases, a statistical comparison was performed to identify DEGs between the libraries using the rigorous algorithm with a false discovery rate (FDR) < 0.001 and an absolute value for the log2 ratio > 1 [65]. To screen for genes involved in the shift between microcycle and normal conidiation, we constructed four subtractive libraries: an SYA vs. SR media library, an SYA vs. NR media library, an SYA vs. PR media library, and an SYA vs. 1/4 SDAY media library.

To identify genes involved in the regulation of the conidiation pattern shift in response to single nutrients, three subtractive libraries based on the in silico analysis were constructed to screen for genes that are specifically expressed in the conidiation pattern shift in response to sucrose, nitrate, or phosphate. To screen for genes that are specifically expressed in the conidiation pattern shift in response to sucrose, the genes expressed in the 1/4 SDAY, NR, and PR media libraries were removed from the SYA vs. SR media subtractive library, and the remaining genes were the sucrose-regulated genes that are involved in the conidiation pattern shift. Nitrate- and phosphate-regulated genes involved in the conidiation pattern shift were also identified using this method.

### GO functional and pathway enrichment analysis

GO is a gene functional classification system with three ontologies: molecular function, cellular component, and biological process. In gene expression profiling analysis, a GO enrichment analysis of functional significance uses a hypergeometric test to map all DEGs to terms in the GO database (http://www.geneontology.org/), looking for significantly enriched GO terms in the DEGs compared to the genome background.

Pathway enrichment analysis identifies significantly enriched metabolic pathways or signal transduction pathways in DEGs by comparison with the whole genome background. The formula is the same as that used in the GO analysis; a pathway with a Q value ≤ 0.05 was defined as having a significant enrichment of DEGs. DEGs that are involved in most major biochemical, metabolism, and signal transduction pathways could be identified through the pathway of significant enrichment.

### Verification of the DEGs by expression profiling via multigene concatemers

To verify the DEGs, 217 genes were randomly chosen from the 436 genes whose expression was up-regulated during microcycle conidiation, and *gapdh* (GenBank accession number: EFY84384), a gene encoding glyceraldehyde 3-phosphate dehydrogenase, was used as an internal control. Primer design was performed according to a previous method [52]. All the primers used in this study are listed in Additional file 11. The expression levels of the selected genes were analyzed in all five media (1/4 SDAY, SYA, SR, NR, and PR) using the MgC-GEP method [52], with minor modifications. For each sample, reverse transcription reaction mixtures were followed by polymerase chain reaction (PCR) amplification with 1 μM of a specific forward primer and 1 μM of a specific reverse primer. The PCR conditions consisted of an initial denaturation at 95 °C for 5 min, followed by five cycles of 94 °C for 30 s, 68 °C for 30 s, 72 °C for 30 s, and ending in a single extension cycle of 72 °C for 5 min. All of the 218 PCR products were mixed and concentrated to 200 μl, and then extracted using the QIAquick Gel Extraction kit (Qiagen, Valencia, CA, USA). The extraction products were further amplified by PCR with 1 μM of a universal forward primer and 1 μM of a universal reverse primer. The amplification conditions were as follows: an initial denaturation at 95 °C for 5 min, followed by 28 cycles of 94 °C for 30 s, 53 °C for 30 s, and 72 °C for 30 s, and ending in a single extension cycle of 72 °C for 5 min. The PCR products were extracted with the QIAquick Gel extraction kit and used for quantitative gene expression analysis as described previously [52].

## Abbreviations

ABC, ATP-binding cassette; ATP, Adenosine triphosphate; bZIP, basic leucine zipper domain; cAMP, cyclic adenosine monophosphate; DGE, digital gene expression; GO, Gene Ontology; HLH, helix-loop-helix; hpi, hours post-inoculation; KEGG, Kyoto Encyclopedia of Genes and Genomes; MAP, mitogen-activated protein; MFS, major facilitator superfamily; MgC-GEP, gene expression profiling via multigene concatemers; NR, nitrate-rich medium; PR, phosphate-rich medium; SDAY, Sabouraud’s dextrose agar medium; SR, sucrose-rich medium; SYA, sucrose yeast extract agar medium; TCA, tricarboxylic acid; ZFP, zinc finger protein

## Additional files

Additional file 1: Distribution of total clean tags and distinct clean tags over different tag abundance categories. Numbers in square brackets demonstrate the range of copy numbers for a specific category of tags. For example, “[2, 5]” means the tags in this category have two to five copies. Numbers in parentheses show the total tag copy number for all the tags in that category. (TIF 5755 kb) Additional file 2: Number of genes in each GO category. (XLS 22 kb) Additional file 3: Genes up-regulated expression during normal conidiation. (XLS 64 kb) Additional file 4: The types and numbers of pathways involved in normal conidiation. (XLS 23 kb) Additional file 5: Genes up-regulated during microcycle conidiation process. (XLS 152 kb) Additional file 6: Sucrose-regulate microcycle conidiation genes. (XLS 48 kb) Additional file 7: The types and numbers of pathways involved in the regulation of the conidiation pattern shift with a single nutrient change. (XLS 29 kb) Additional file 8: Nitrate-regulate microcycle conidiation genes. (XLS 83 kb) Additional file 9: Phosphate-regulate microcycle conidiation genes. (XLS 71 kb) Additional file 10: Genes used for expression profiling via multigene concatemers. (XLS 123 kb) Additional file 11: Primers for MgC-GEP and genes verified by MgC-GEP. (XLS 79 kb)

## References

[1] Nielsen J (1992). Modelling the growth of filamentous fungi. Adv Biochem Eng Biotechnol.

[2] Papagianni M (2004). Fungal morphology and metabolite production in submerged mycelial processes. Biotechnol Adv.

[3] Hanlin R (1994). Microcycle conidiation — A review. Mycoscience.

[4] Park HS, Yu JH (2012). Genetic control of asexual sporulation in filamentous fungi. Curr Opin Microbiol.

[5] Ahearn DG, Price D, Simmons RB, Mavo A, Zhang ST, Crow SA (2007). Microcycle conidiation and medusa head conidiophores of aspergilli on indoor construction materials and air filters from hospitals. Mycologia.

[6] Anderson JG, Smith JE (1971). The production of conidiophores and conidia by newly germinated conidia of *Aspergillus niger* (microcycle conidiation). J Gen Microbiol.

[7] Lapaire CL, Dunkle LD (2003). Microcycle conidiation in *Cercospora zeae-maydis*. Phytopathology.

[8] Maheshwari R (1999). Microconidia of *Neurospora crassa*. Fungal Genet Biol.

[9] Jung B, Kim S, Lee J (2014). Microcyle conidiation in filamentous fungi. Mycology.

[10] Sekiguchi J, Gaucher GM, Costerton JW (1975). Microcycle conidiation in *Penicillium urticae*: an ultrastructural investigation of conidiogenesis. Can J Microbiol.

[11] Mieslerova B, Lebeda A (2010). Influence of temperature and light conditions on germination, growth and conidiation of *Oidium neolycopersici*. J Phytopathol.

[12] Pažout J, Schröder P (1988). Microcycle conidiation in submerged cultures of *Penicillium cyclopium* attained without temperature changes. J Gen Microbiol.

[13] Liu Q, Xiao CL (2005). Influence of nutrient and environmental factors on conidial germination of *Potebniamyces pyri*. Phytopathology.

[14] Vezina C, Singh K, Sehgal SN (1965). Sporulation of filamentous fungi in submerged culture. Mycologia.

[15] Bosch A, Yantorno O (1999). Microcycle conidiation in the entomopathogenic fungus *Beauveria bassiana* bals. (vuill.). Process Biochem.

[16] Rangel D, Braga G, Anderson A, Roberts D (2005). Influence of growth environment on tolerance to uv-b radiation, germination speed, and morphology of *Metarhizium anisopliae* var. *acridum* conidia. J Invertebr Pathol.

[17] Cascino JJ, Harris RF, Smith CS, Andrews JH (1990). Spore yield and microcycle conidiation of *Colletotrichum gloeosporioides* in liquid culture. Appl Environ Microbiol.

[18] Khurana N, Saxena RK, Gupta R, Rajam MV (1996). Polyamines as modulators of microcycle conidiation in *Aspergillus flavus*. Microbiology.

[19] Pandit A, Maheshwari R (1996). Life-history of *Neurospora intermedia* in a sugar cane field. J Bioscience.

[20] Osherov N, May GS (2001). The molecular mechanisms of conidial germination. FEMS Microbiol Lett.

[21] Furlong MJ, Pell JK (1996). Interactions between the fungal entomopathogen *Zoophthora radicans* Brefeld (Entomophthorales) and two hymenopteran parasitoids attacking the diamondback moth, *Plutella xylostella* L. J Invertebr Pathol.

[22] Lord JC (2001). Response of the wasp *Cephalonomia tarsalis* (Hymenoptera : Bethylidae) to *Beauveria bassiana* (Hyphomycetes : Moniliales) as free conidia or infection in its host, the sawtoothed grain beetle, *Oryzaephilus surinamensis* (Coleoptera : Silvanidae). Bio Control.

[23] Nielsen C, Skovgard H, Steenberg T (2005). Effect of *Metarhizium anisopliae* (Deuteromycotina : Hyphomycetes) on survival and reproduction of the filth fly parasitoid, *Spalangia cameroni* (Hymenoptera : Pteromalidae). Environ Entomol.

[24] Hunter DM, Milner RJ, Spurgin PA (2001). Aerial treatment of the Australian plague locust, *Chortoicetes terminifera* (Orthoptera: Acrididae) with *Metarhizium anisopliae* (Deuteromycotina: Hyphomycetes). Bull Entomol Res.

[25] Peng GX, Wang ZK, Yin YP, Zeng DY, Xia YX (2008). Field trials of *Metarhizium anisopliae* var. *acridum* (Ascomycota : Hypocreales) against oriental migratory locusts, *Locusta migratoria manilensis* (Meyen) in Northern China. Crop Prot.

[26] Li Z, Alves S, Roberts D, Fan M, Delalibera I, Tang J (2010). Biological control of insects in Brazil and China: history, current programs and reasons for their successes using entomopathogenic fungi. Biocontrol Sci Techn.

[27] Zhang SZ, Peng GX, Xia YX (2010). Microcycle conidiation and the conidial properties in the entomopathogenic fungus *Metarhizium acridum* on agar medium. Biocontrol Sci Techn.

[28] Gao Q, Jin K, Ying SH, Zhang YJ, Xiao GH, Shang YF (2011). Genome sequencing and comparative transcriptomics of the model entomopathogenic fungi Metarhizium anisopliae and M. acridum. PLoS Genet.

[29] Saito RM, Perreault A, Peach B, Satterlee JS, van den Heuvel S (2004). The CDC-14 phosphatase controls developmental cell-cycle arrest in *C. elegans*. Nat Cell Biol.

[30] Huang SM, Schonthal AH, Stallcup MR (2001). Enhancement of p53-dependent gene activation by the transcriptional coactivator Zac1. Oncogene.

[31] Loudet O, Michael TP, Burger BT, Le Mette C, Mockler TC, Weigel D (2008). A zinc knuckle protein that negatively controls morning-specific growth in *Arabidopsis thaliana*. P Natl Acad Sci USA.

[32] Morita H, Tomita S, Maeda H, Okamoto A, Yamagata Y, Kusumoto K (2012). Serine-type carboxypeptidase KexA of *Aspergillus oryzae* has broader substrate specificity than *Saccharomyces cerevisiae* Kex1 and is required for normal hyphal growth and conidiation. Appl Environ Microbiol.

[33] Miyagawa Y, Tamoi M, Shigeoka S (2001). Overexpression of a cyanobacterial fructose-1,6-/sedoheptulose-1,7-bisphosphatase in tobacco enhances photosynthesis and growth. Nat Biotechnol.

[34] Hansberg W, Salas-Lizana R, Dominguez L (2012). Fungal catalases: function, phylogenetic origin and structure. Arch Biochem Biophys.

[35] Shibuya K, Paris S, Ando T, Nakayama H, Hatori T, Latge JP (2006). Catalases of *Aspergillus fumigatus* and inflammation in aspergillosis. Nihon Ishinkin Gakkai Zasshi.

[36] Gonzalez FJ (2005). Role of cytochromes P450 in chemical toxicity and oxidative stress: studies with CYP2E1. Mutat Res.

[37] Kitagaki H, Wu H, Shimoi H, Ito K (2002). Two homologous genes, *DCW1* (*YKL046c*) and *DFG5*, are essential for cell growth and encode glycosylphosphatidylinositol (GPI)-anchored membrane proteins required for cell wall biogenesis in *Saccharomyces cerevisiae*. Mol Microbiol.

[38] Montijn RC, van Rinsum J, van Schagen FA, Klis FM (1994). Glucomannoproteins in the cell wall of *Saccharomyces cerevisiae* contain a novel type of carbohydrate side chain. J Biol Chem.

[39] Freeman NL, Lila T, Mintzer KA, Chen Z, Pahk AJ, Ren R (1996). A conserved proline-rich region of the *Saccharomyces cerevisiae* cyclase-associated protein binds SH3 domains and modulates cytoskeletal localization. Mol Cell Biol.

[40] Sauve S, Naud JF, Lavigne P (2007). The mechanism of discrimination between cognate and non-specific DNA by dimeric b/HLH/LZ transcription factors. J Mol Biol.

[41] Roze LV, Beaudry RM, Keller NP, Linz JE (2004). Regulation of aflatoxin synthesis by FadA/cAMP/protein kinase A signaling in *Aspergillus parasiticus*. Mycopathologia.

[42] Brodhagen M, Keller NP (2006). Signalling pathways connecting mycotoxin production and sporulation. Mol Plant Pathol.

[43] Roncal T, Ugalde U (2003). Conidiation induction in *Penicillium*. Res Microbiol.

[44] Yuan H, Liu D (2008). Signaling components involved in plant responses to phosphate starvation. J Integr Plant Biol.

[45] Kronstad J, De Maria A, Funnell D, Laidlaw RD, Lee N, Ramesh MMDM (1998). Signaling via cAMP in fungi: interconnections with mitogen-activated protein kinase pathways. Arch Microbiol.

[46] Springer J, Kneissl M, Putter V, Grummt F (1999). Identification and characterization of MmORC4 and MmORC5, two subunits of the mouse origin of replication recognition complex. Chromosoma.

[47] Ruizperez VL, Murillo FJ, Torresmartinez S (1995). PkpA, a Novel *Phycomyces-Blakesleeanus* serine/threonine protein kinase. Curr Genet.

[48] Pera LM, Baigorí MD, Callieri D (1999). Influence of environmental conditions on hyphal morphology in pellets of *Aspergillus niger*: role of β-N-acetyl-D-glucosaminidase. Curr Microbiol.

[49] Kamisaka Y, Yokochi T, Nakahara T, Suzuki O (1993). Characterization of the diacylglycerol acyltransferase activity in the membrane-fraction from a fungus. Lipids.

[50] Geckil H, Gencer S (2004). Production of L-asparaginase in *Enterobacter aerogenes* expressing *Vitreoscilla* hemoglobin for efficient oxygen uptake. Appl Microbiol Biot.

[51] Qin M, Zhao FS (2003). L-asparaginase release from *Escherichia coli* cells with aqueous two-phase micellar systems. Appl Biochem Biotech.

[52] Jin K, Zheng X, Xia Y (2011). Gene expression profiling via multigene concatemers. PLoS One.

[53] Liu B, Morris NR (2000). A spindle pole body-associated protein, SNAD, affects septation and conidiation in Aspergillus nidulans. Mol Gen Genet.

[54] Kays AM, Rowley PS, Baasiri RA, Borkovich KA (2000). Regulation of conidiation and adenylyl cyclase levels by the Galpha protein GNA-3 in *Neurospora crassa*. Mol Cell Biol.

[55] Yang M, Jin K, Xia YX (2011). MaFKS, a beta-1,3-glucan synthase, is involved in cell wall integrity, hyperosmotic pressure tolerance and conidiation in *Metarhizium acridum*. Curr Genet.

[56] Slade SJ, Harris RF, Smith CS, Andrews JH (1987). Microcycle conidiation and spore-carrying capacity of *Colletotrichum gloeosporioides* on solid media. Appl Environ Microb.

[57] Ebbole DJ (1998). Carbon catabolite repression of gene expression and conidiation in Neurospora crassa. Fungal Genet Biol.

[58] Fisher SH (1999). Regulation of nitrogen metabolism in *Bacillus subtilis*: vive la difference!. Mol Microbiol.

[59] Khurana N, Saxena RK, Gupta R, Kuhad RC (1993). Light-independent conidiation in *Trichoderma* spp. a novel approach to microcycle conidiation. World J Microb Biot.

[60] Maheshwari R (1991). Microcycle conidiation and its genetic-basis in *Neurospora crassa*. J Gen Microbiol.

[61] Huo Z, Yang X, Raza W, Huang Q, Xu Y, Shen Q (2010). Investigation of factors influencing spore germination of *Paenibacillus polymyxa* ACCC10252 and SQR-21. Appl Microbiol Biotechnol.

[62] Xu X, Yang J, An Y, Pan Y, Liu G (2012). Over-expression of *pcvA* involved in vesicle-vacuolar fusion affects the conidiation and penicillin production in Penicillium chrysogenum. Biotechnol Lett.

[63] Zuber S, Hynes MJ, Andrianopoulos A (2002). G-protein signaling mediates asexual development at 25 degrees C but has no effect on yeast-like growth at 37 degrees C in the dimorphic fungus *Penicillium marneffei*. Eukaryot Cell.

[64] Zhang S, Xia Y (2008). Identification of genes preferentially expressed during microcycle conidiation of *Metarhizium anisopliae* using suppression subtractive hybridization. FEMS Microbiol Lett.

[65] Audic S, Claverie JM (1997). The significance of digital gene expression profiles. Genome Res.

[66] Dünkler A, Jorde S, Wendland J (2008). An *Ashbya gossypii cts2* mutant deficient in a sporulation-specific chitinase can be complemented by *Candida albicans CHT4*. Microbiol Res.

[67] Yamashita N, Motoyoshi T, Nishimura A (2000). Molecular cloning of the isoamyl alcohol oxidase-encoding gene (*mreA*) from *Aspergillus oryzae*. J Biosci Bioeng.

[68] Laity JH, Lee BM, Wright PE (2001). Zinc finger proteins: new insights into structural and functional diversity. Curr Opin Struct Biol.

[69] Gołda A, Szyniarowski P, Ostrowska K, Kozik A, Rapała-Kozik M (2004). Thiamine binding and metabolism in germinating seeds of selected cereals and legumes. Plant Physiol Biochem.

[70] Bhoumik A, Lopez-Bergami P, Ronai Z (2007). ATF2 on the double-activating transcription factor and DNA damage response protein. Pigment Cell Res.

[71] Jeney A, Béki E, Keszthelyi A, Leslie JF, Hornok L (2007). Cloning and characterization of *Fpmtr1*, an amino acid transporter gene of *Fusarium proliferatum* (*Gibberella intermedia*). J Basic Microbiol.

[72] Giefing-Kröll C, Jelencsics KE, Reipert S, Nagy E (2011). Absence of pneumococcal PcsB is associated with overexpression of LysM domain-containing proteins. Microbiology.

[73] Pistorius S, Goergens H, Engel C, Plaschke J, Krueger S, Hoehl R (2007). N-Acetyltransferase (NAT) 2 acetylator status and age of tumour onset in patients with sporadic and familial, microsatellite stable (MSS) colorectal cancer. Int J Colorectal Dis.

[74] Arias CA, Panesso D, McGrath DM, Qin X, Mojica MF, Miller C (2011). Genetic basis for *in vivo* daptomycin resistance in enterococci. N Engl J Med.

[75] Sevier CS (2012). Erv2 and quiescin sulfhydryl oxidases: Erv-domain enzymes associated with the secretory pathway. Antioxid Redox Sign.

[76] Feng L, Wang K, Li Y, Tan Y, Kong J, Li H (2007). Overexpression of SBPase enhances photosynthesis against high temperature stress in transgenic rice plants. Plant Cell Rep.

[77] Mathews WR, Wang F, Eide DJ, Doren MV (2005). *Drosophila* fear of intimacy encodes a Zrt/IRT-like protein (ZIP) family zinc transporter functionally related to mammalian ZIP proteins. J Biol Chem.

[78] Carrozza MJ, Utley RT, Workman JL, Cote J (2003). The diverse functions of histone acetyltransferase complexes. Trends Genet.

[79] Nakayama K, Nakayama N, Davidson B, Sheu JJ, Jinawath N, Santillan A (2006). A BTB/POZ protein, NAC-1, is related to tumor recurrence and is essential for tumor growth and survival. Proc Natl Acad Sci.

[80] Abramyan J, Feng CW, Koopman P (2009). Cloning and expression of candidate sexual development genes in the cane toad (*Bufo marinus*). Dev Dyn.

[81] Hagemann C, Weigelin B, Schommer S, Schulze M, Al-Jomah N, Anacker J (2011). The cohesin-interacting protein, precocious dissociation of sisters 5A/sister chromatid cohesion protein 112, is up-regulated in human astrocytic tumors. Int J Mol Med.

[82] Ahn JD, Morishita R, Kaneda Y, Lee SJ, Kwon KY, Choi SY (2002). Inhibitory effects of novel AP-1 decoy oligodeoxynucleotides on vascular smooth muscle cell proliferation in vitro and neointimal formation *in vivo*. Circ Res.

[83] Chekanova JA, Shaw RJ, Wills MA, Belostotsky DA (2000). Poly(A) tail-dependent exonuclease AtRrp41p from *Arabidopsis thaliana* rescues 5.8 S rRNA processing and mRNA decay defects of the yeast *ski6* mutant and is found in an exosome-sized complex in plant and yeast cells. J Biol Chem.

[84] Zuo Z, Urban G, Scammell JG, Dean NM, McLean TK, Aragon I (1999). Ser/Thr protein phosphatase type 5 (PP5) is a negative regulator of glucocorticoid receptor‐mediated growth arrest. Biochemistry.

[85] Passalacqua M, Patrone M, Picotti GB, Del Rio M, Sparatore B, Melloni E (1998). Stimulated astrocytes release high-mobility group 1 protein, an inducer of LAN-5 neuroblastoma cell differentiation. Neuroscience.

[86] Guizzetti M, Costa LG (1996). Inhibition of muscarinic receptor-stimulated glial cell proliferation by ethanol. J Neurochem.

[87] Kunzelmann K (2005). Ion channels and cancer. J Membr Biol.

[88] Boughton BA, Dobson RC, Gerrard JA, Hutton CA (2008). Conformationally constrained diketopimelic acid analogues as inhibitors of dihydrodipicolinate synthase. Bioorg Med Chem Lett.

[89] Kershaw MJ, Thornton CR, Wakley GE, Talbot NJ (2005). Four conserved intramolecular disulphide linkages are required for secretion and cell wall localization of a hydrophobin during fungal morphogenesis. Mol Microbiol.

[90] Xiao Z, Xue J, Sowin TJ, Zhang H (2006). Differential roles of checkpoint kinase 1, checkpoint kinase 2, and mitogen-activated protein kinase-activated protein kinase 2 in mediating DNA damage-induced cell cycle arrest: implications for cancertherapy. Mol Cancer Ther.

[91] Jouany JP, Medina B, Bertin G, Julliand V (2009). Effect of live yeast culture supplementation on hindgut microbial communities and their polysaccharidase and glycoside hydrolase activities in horses fed a high-fiber or high-starch diet. J Anim Sci.

[92] de Cárcer G, Pérez de Castro I, Malumbres M (2007). Targeting cell cycle kinases for cancer therapy. Curr Med Chem.

[93] Schneiter R, Toulmay A (2007). The role of lipids in the biogenesis of integral membrane proteins. Appl Microbiol Biotechnol.

[94] Jover R, Hoffmann F, Scheffler-Koch V, Lindberg RL (2000). Limited heme synthesis in porphobilinogen deaminase-deficient mice impairs transcriptional activation of specific cytochrome P450 genes by phenobarbital. Eur J Biochem.

[95] Chan MT, Yu SM (1998). The 39 untranslated region of a rice alpha-amylase gene functions as a sugar-dependent mRNA stability determinant. Proc Natl Acad Sci.

[96] Vierheilig H, Alt-Hug M, Wiemken A, Boller T (2001). Hyphal in vitro growth of the arbuscular mycorrhizal fungus *Glomus mosseae* is affected by chitinase but not by β-1,3-glucanase. Mycorrhiza.

[97] Van der Meer JR, Frijters AC, Leveau JH, Eggen RI, Zehnder AJ, de Vos WM (1991). Characterization of the *Pseudomonas* sp. strain P51 gene *tcbR*, a LysR-type transcriptional activator of the *tcbCDEF* chlorocatechol oxidative operon, and analyses of the regulatory region. J Bacteriol.

[98] Iaaska VK, Iaaska BE (1978). Electrophoretic analysis of substrate specificity of wheat alcohol dehydrogenases. Biokhimiia.

[99] Shimizu M, Goto M, Hanai M, Shimizu T, Izawa N, Kanamoto H (2008). Selectable tolerance to herbicides by mutated acetolactate synthase gees integrated into the chloroplast genome of tobacco. Plant Physiol.

