# Supplementary figures and images for: Transcriptional analysis of the conidiation pattern shift of the entomopathogenic fungus Metarhizium acridum in response to different nutrients
Source: BMC Genomics. 2016 Aug 9;17:586. doi: 10.1186/s12864-016-2971-0 (PMC4979188; doi:10.1186/s12864-016-2971-0)

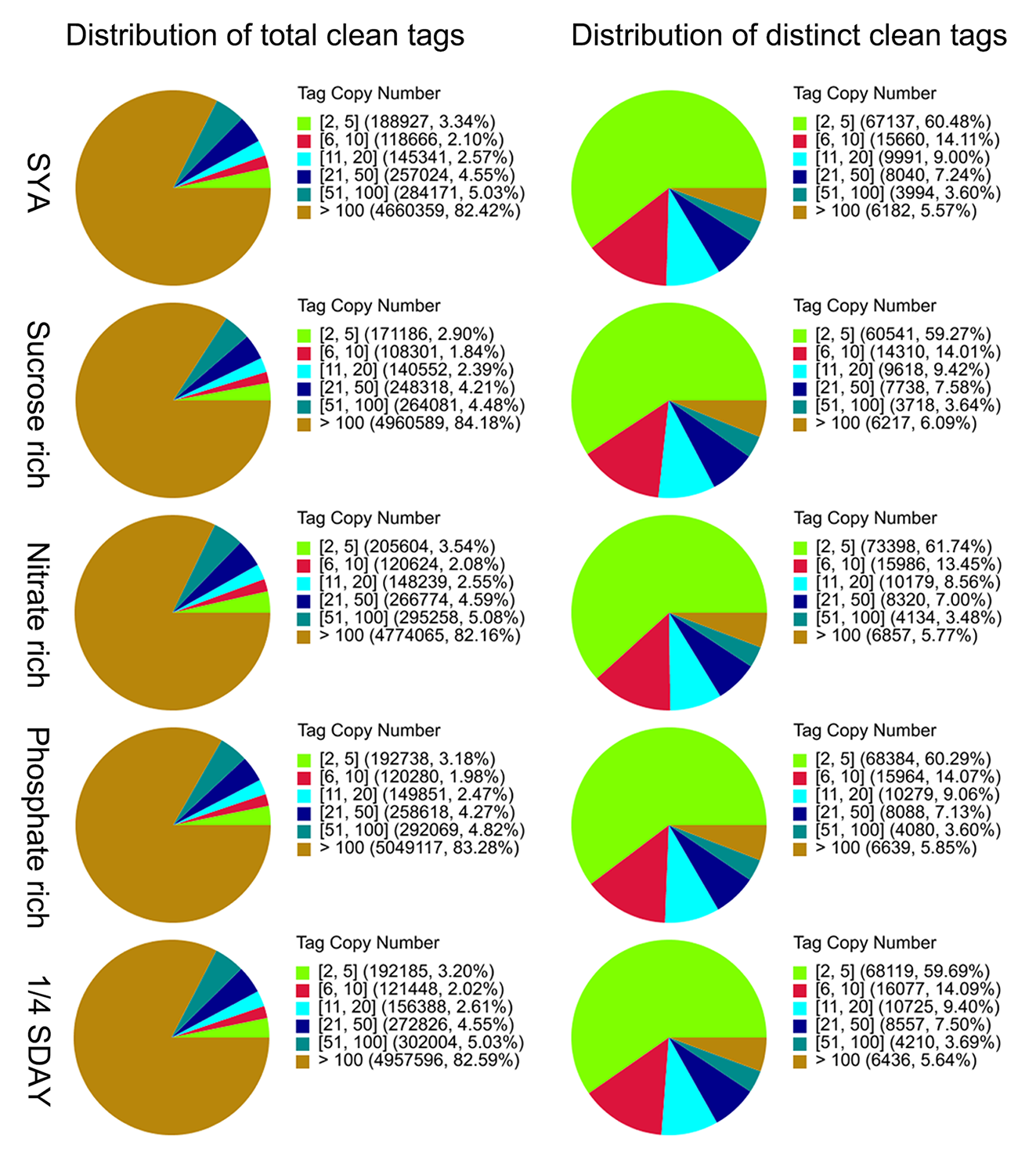

Supplement: Additional file 1: — Distribution of total clean tags and distinct clean tags over different tag abundance categories. Numbers in square brackets demonstrate the range of copy numbers for a specific category of tags. For example, “[2, 5]” means the tags in this category have two to five copies. Numbers in parentheses show the total tag copy number for all the tags in that category. (TIF 5755 kb) [file 12864_2016_2971_MOESM1_ESM.tif]
